# Scarlet Fever by Mail

**Published:** 1875-04

**Authors:** 


SCARLET FEVER BY MAIL.—Mr. J. W. Land, of Exmouth, England, writes to the *Lancet* as follows: "As a small contribution to the correspondence now going on in your columns on the subject of scarlatina, I send you this, as showing how it may be transmitted from one part of the kingdom to another. In the spring of last year I received a newspaper from Inverness containing the announcement of the death of a friend's child. About a week afterwards the first symptoms of scarlatina showed themselves, and I suffered a mild (second) attack of the disease. There being no other case either in my own practice or in that of all the neighboring practitioners at the time, I was rather puzzled to account for it. Within the last few days I have heard that my friend's child's death was caused by a most malignant attack of scarlet fever, (the attendant ordering burial the next day), which infected nearly all the rest of the household."

